# Supplementary material for: Lipidated peptides derived from intracellular loops 2 and 3 of the urotensin II receptor act as biased allosteric ligands
Source: J Biol Chem. 2021 Aug 10;297(3):101057. doi: 10.1016/j.jbc.2021.101057 (PMC8424217; doi:10.1016/j.jbc.2021.101057)
Supplement: Figures S1 and S2; and Tables S1–S6 [file mmc1.pdf]

Membrane-tethered peptides derived from intracellular loops 2 and 3 of the urotensin II receptor act as allosteric biased ligands

Hassan Nassour[1,†], Tuan Anh Hoang[1,†], Ryan D. Martin[2], Juliana C. Dallagnol[1,2,3], Étienne Billard[1], Létourneau Myriam[1], Ettore Novellino[3], Alfonso Carotenuto[3], Bruce G. Allen[4], Jason C. Tanny[2], Alain Fournier[1], Terence E. Hébert[2], and David Chatenet[1*]

Running Title: *hUT-derived pepducins enables urotensin II receptor activation*

Supplementary Information

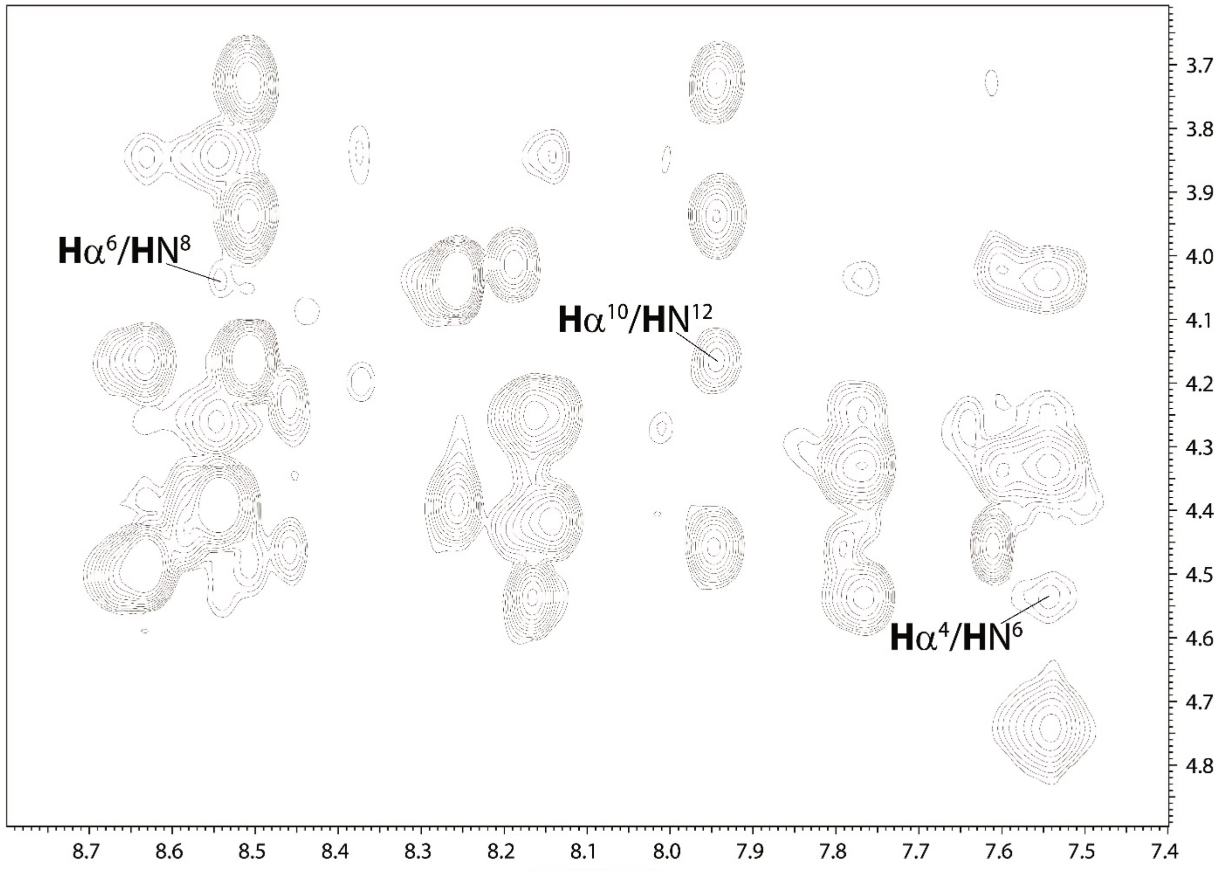

Figure S1. Expanded region of the NOESY spectrum of hUT-Pep2. Diagnostic NOEs are highlighted.

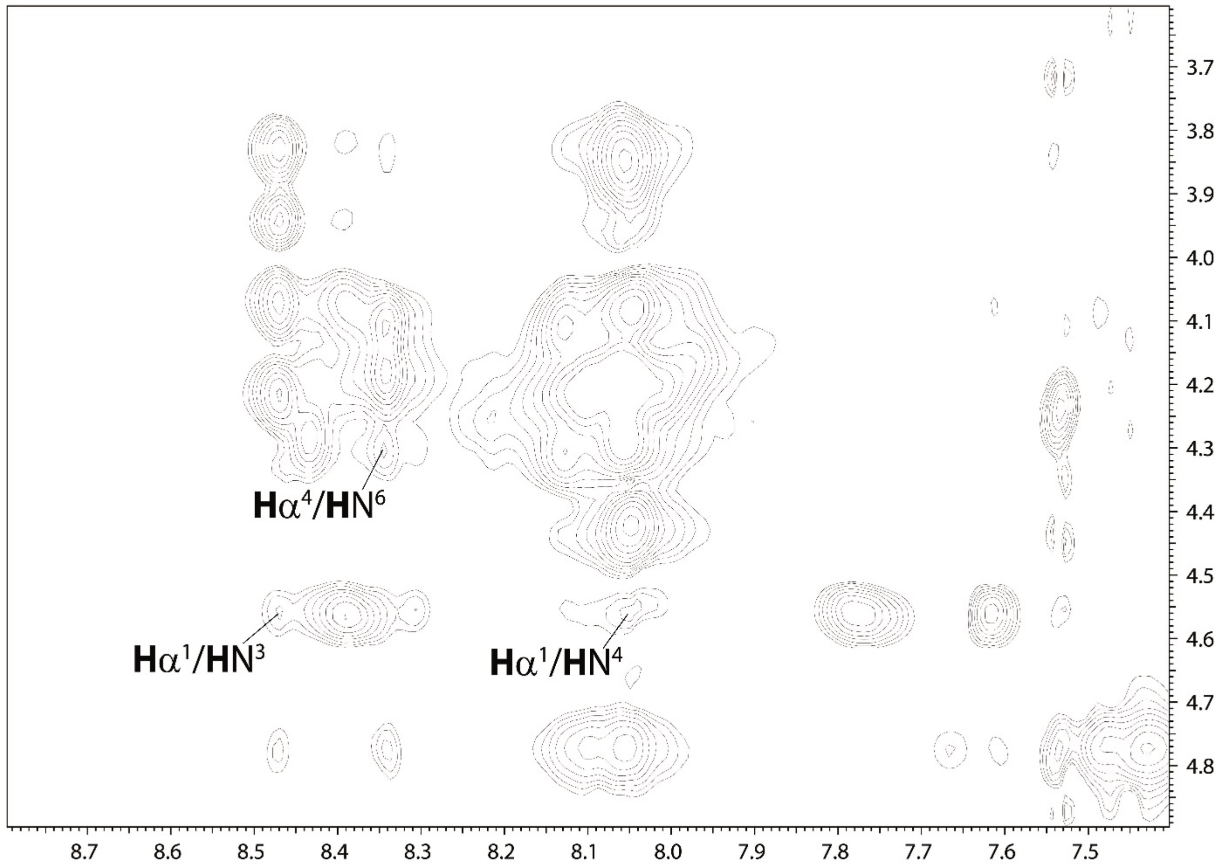

Figure S2. Expanded region of the NOESY spectrum of [Trp[1], Leu[2]]hUT-Pep3. Diagnostic NOEs are highlighted.

Table S1. Resonance Assignments[a] of hUT-Pep2 in DPC solution at 25°C

Residue	NH ($^3J_{\alpha N}$, -Δδ/ΔT)[b]	$C^{\alpha}H$ (Δδ rc)[c]	$C^{\beta}H$	Others
Arg[1]	8.55 (nd; nd)	4.25 (-0.09)	1.83	3.22(δ); 7.63(ε); 1.66, 1.69(δ)
Pro[2]		4.41 (-0.02)	2.29; 2.39	1.99(γ); 3.40, 3.84(δ)
Leu[3]	8.14 (5.2; 5.8)	4.24 (-0.08)	1.78	1.66(γ); 0.85, 0.92(δ)
Asp[4]	8.16 (4.9; 2.7)	4.54 (-0.11)	2.81	
Thr[5]	7.77 (bs; 2.0)	4.33 (-0.04)		1.17(γ)
Val[6]	7.54 (ov; 0.3)	4.03 (-0.07)	2.13	0.92, 0.97(γ)
Gln[7]	8.26 (5.6; 8.0)	4.39 (0.05)	1.91, 2.03	2.32(γ); 6.82, 7.57(ε)
Arg[8]	8.54 (6.4; 11.0)	4.39 (-0.24)	1.76	1.66(γ); 3.16(δ); 7.49(ε)
Pro[9]		4.48 (0.05)	2.35, 1.92	2.03(γ) ; 3.44, 3.84(δ)
Lys[10]	8.63 (bs; 10.5)	4.16 (-0.16)	1.91, 1.77	1.41, 1.48(γ); 1.66(δ); 2.97(ε); 7.54(ζ)
Gly[11]	8.51 (5.4; 10.1)	3.74, 3.94 (-012)		
Tyr[12]	7.94 (6.6;4.9)	4.45 (-0.11)	2.83, 3.02	7.01(δ); 6.76(ε)

[a] Obtained with TSP (δ 0.00 ppm) as reference shift. Chemical shifts are accurate to ±0.02 ppm.
[b] $^3J_{\alpha N}$ coupling constants in Hz. -Δδ/ΔT = temperature coefficients (ppb/K) calculated in the range 25-35 °C. [c] Random coil values from: Andersen et al. FEBS Lett. 1996, 399, 47-52. Further signals: C-terminal amide group, 7.08, 7.61 ppm; palmitic acid, 2.30, 1.55, 1.27-1.18, 0.77 ppm. nd: not determined; bs: broad signal; ov: overlapped signal.

Table S2. NOE derived Upper Limit Constraints (Å) of hUT-Pep2.

1	ARG	HN	2	PRO	QD	5.34
1	ARG	HA	1	ARG	HG2	3.61
1	ARG	HA	1	ARG	HG3	3.61
1	ARG	HA	1	ARG	QG	3.15
1	ARG	HA	1	ARG	QD	5.88
1	ARG	HA	2	PRO	HD2	3.86
1	ARG	HA	2	PRO	HD3	3.86
1	ARG	HA	2	PRO	QD	3.30
1	ARG	QB	1	ARG	HE	6.16
2	PRO	HA	3	LEU	HN	3.41
2	PRO	HA	5	THR	QG2	6.28
2	PRO	QB	3	LEU	HN	3.77
2	PRO	HD2	3	LEU	HN	5.50
2	PRO	HD3	3	LEU	HN	5.50
2	PRO	QD	3	LEU	HN	4.75
3	LEU	HN	3	LEU	HG	3.11
3	LEU	HN	3	LEU	QD1	5.23
3	LEU	HN	3	LEU	QD2	5.23
3	LEU	HN	3	LEU	QQD	4.58
3	LEU	HA	3	LEU	HG	2.74
3	LEU	HA	3	LEU	QD1	4.36
3	LEU	HA	3	LEU	QD2	4.36
3	LEU	HA	3	LEU	QQD	3.87
3	LEU	HA	4	ASP	QB	5.04
3	LEU	HA	5	THR	HN	3.86
3	LEU	HA	6	VAL	HN	4.20
3	LEU	HA	6	VAL	HB	3.83
3	LEU	HA	6	VAL	QQG	4.51
4	ASP	HN	4	ASP	QB	4.02
4	ASP	HN	5	THR	HN	3.24
4	ASP	HA	5	THR	HN	3.33
4	ASP	HA	6	VAL	HN	4.45
4	ASP	QB	5	THR	HN	5.04
4	ASP	QB	8	ARG	QG	7.25
4	ASP	QB	8	ARG	QD	7.25
5	THR	HN	5	THR	QG2	4.76
5	THR	HN	6	VAL	HN	3.11
5	THR	HN	6	VAL	QQG	5.92
5	THR	HA	5	THR	QG2	3.83
5	THR	HA	6	VAL	HN	3.05
5	THR	HA	7	GLN	HN	4.32
5	THR	QG2	6	VAL	HN	6.53
6	VAL	HN	6	VAL	HB	3.21
6	VAL	HN	6	VAL	QG1	5.07
6	VAL	HN	6	VAL	QG2	5.07
6	VAL	HN	6	VAL	QQG	4.12
6	VAL	HN	7	GLN	HN	3.64
6	VAL	HA	6	VAL	HB	3.02
6	VAL	HA	6	VAL	QG1	4.02
6	VAL	HA	6	VAL	QG2	4.02
6	VAL	HA	7	GLN	HN	3.49
6	VAL	HA	8	ARG	HN	4.57
6	VAL	HB	7	GLN	HN	3.33
6	VAL	QG1	7	GLN	HN	5.04
6	VAL	QG1	7	GLN	QG	7.40

6	VAL	QG2	7	GLN	HN	5.04
6	VAL	QG2	7	GLN	QG	7.40
6	VAL	QQG	7	GLN	HN	4.17
7	GLN	HN	7	GLN	HB2	3.27
7	GLN	HN	7	GLN	HB3	3.27
7	GLN	HN	7	GLN	QB	2.77
7	GLN	HN	7	GLN	QG	5.20
7	GLN	HN	8	ARG	HN	3.79
7	GLN	HA	7	GLN	HB2	3.05
7	GLN	HA	7	GLN	HB3	3.05
7	GLN	HA	7	GLN	QB	2.62
7	GLN	HB2	8	ARG	HN	3.52
7	GLN	HB3	8	ARG	HN	3.52
8	ARG	HN	8	ARG	QB	4.18
8	ARG	HN	8	ARG	QG	4.92
8	ARG	HN	8	ARG	QD	6.38
8	ARG	HN	9	PRO	QB	5.35
8	ARG	HN	9	PRO	QG	5.88
8	ARG	HN	9	PRO	QD	4.38
8	ARG	HA	8	ARG	QD	6.00
8	ARG	HA	9	PRO	HD2	3.02
8	ARG	HA	9	PRO	HD3	3.02
8	ARG	QB	8	ARG	HE	6.04
8	ARG	QB	9	PRO	QD	4.78
8	ARG	QG	9	PRO	QD	5.36
8	ARG	QD	13	CNH2	HN2	6.38
9	PRO	HA	10	LYS	HN	2.59
9	PRO	QB	10	LYS	HN	4.70
9	PRO	QB	12	TYR	QD	8.52
9	PRO	QB	12	TYR	QE	8.51
9	PRO	QG	10	LYS	HN	5.60
9	PRO	QG	12	TYR	HB2	6.38
9	PRO	HD2	10	LYS	HN	5.50
9	PRO	HD2	12	TYR	HB2	5.50
9	PRO	HD2	12	TYR	QD	7.64
9	PRO	HD3	10	LYS	HN	5.50
9	PRO	HD3	12	TYR	HB2	5.50
9	PRO	HD3	12	TYR	QD	7.64
9	PRO	QD	12	TYR	HB2	4.84
9	PRO	QD	12	TYR	HB3	5.34
10	LYS	HN	10	LYS	QB	3.90
10	LYS	HN	10	LYS	HG2	4.91
10	LYS	HN	10	LYS	HG3	4.91
10	LYS	HN	10	LYS	QG	4.22
10	LYS	HN	10	LYS	QD	6.38
10	LYS	HN	11	GLY	HN	3.58
10	LYS	HA	10	LYS	HG2	4.07
10	LYS	HA	10	LYS	HG3	4.07
10	LYS	HA	10	LYS	QG	3.57
10	LYS	HA	10	LYS	QD	5.79
10	LYS	HA	10	LYS	QE	6.38
10	LYS	HA	11	GLY	HN	2.62
10	LYS	HA	12	TYR	HN	3.76
10	LYS	QB	10	LYS	QE	7.25
10	LYS	QB	10	LYS	QZ	7.32
10	LYS	QB	11	GLY	HN	4.55
10	LYS	HG2	11	GLY	HN	5.50

10	LYS	HG3	11	GLY	HN	5.50
10	LYS	QD	11	GLY	HN	6.38
11	GLY	HN	11	GLY	HA1	2.93
11	GLY	HN	11	GLY	HA2	2.93
11	GLY	HN	11	GLY	QA	2.55
11	GLY	HN	12	TYR	HN	3.14
11	GLY	HN	12	TYR	QD	7.64
11	GLY	HN	13	CNH2	HN1	5.50
11	GLY	HA1	12	TYR	HN	3.33
11	GLY	HA1	12	TYR	QD	7.64
11	GLY	HA2	12	TYR	HN	3.33
11	GLY	HA2	12	TYR	QD	7.64
12	TYR	HN	12	TYR	HB2	2.90
12	TYR	HN	12	TYR	HB3	3.21
12	TYR	HN	12	TYR	QD	6.40
12	TYR	HN	12	TYR	QE	7.63
12	TYR	HN	13	CNH2	HN1	4.07
12	TYR	HN	13	CNH2	HN2	5.07
12	TYR	HA	12	TYR	HB3	2.99
12	TYR	HA	12	TYR	QD	6.15
12	TYR	HB2	13	CNH2	HN1	5.25
12	TYR	HB3	13	CNH2	HN1	4.91
12	TYR	HB3	13	CNH2	HN2	5.34

Table S3. Resonance Assignments[a] of peptide [Trp^1, Leu^2]hUT-Pep3 in DPC solution at 25°C

Residue	NH ($^3J_{\alpha N}$, -Δδ/ΔT)[b]	C$^\alpha$H (Δδ rc)[c]	C$^\beta$H	Others
Trp^1	7.77 (bs ; 7.1)	4.55 (-0.10)	3.29, 3.38	7.32(δ); 7.61(ε); 6.96, 7.46 (ζ); 7.06 (η)
Leu^2	8.39 (bs; 12.7)	4.06 (-0.26)	1.50; 1.56	1.62(γ); 0.83, 0.86(δ)
Ser^3	8.46 (4.9; 7.3)	4.21 (-0.26)	3.82, 3.93	
Gln^4	8.05 (ov; -3.5)	4.30 (-0.04)	2.11	2.35(γ); 6.73, 7.53(ε)
Arg^5	8.12 (bs ; 4.1)	4.10 (-0.24)	1.85	1.65(γ); 3.12, 3.20(δ); 7.47(ε)
Ala^6	8.34 (bs; 6.7)	4.17 (-0.15)	1.41	
Ser^7	8.05 (nd; 2.9)	4.27 (-0.20)	3.82, 3.86	
Phe^8	8.05 (bs; 2.6)	4.42 (-0.19)	3.10, 3.21	7.22(δ)
Lys^9	8.04 (bs; 3.0)	4.07 (-0.25)	1.83	1.41, 1.52(γ) ; 1.69(δ); 2.95(ε); 7.66 (ζ);
Arg^{10}	8.04 (ov; 0.6)	4.21 (-0.14)	1.71, 1.83	1.59(γ); 3.11(δ); 7.42(ε)
Ala^{11}	8.09 (ov; 1.3)	4.21 (-0.11)	1.38	
Arg^{12}	8.10 (ov; 3.7)	4.16 (-0.18)	1.79, 1.87	1.65, 1.69(γ); 3.19(δ); 7.43(ε)
Arg^{13}	8.14 (bs; 1.3)	4.24 (-0.10)	1.79, 1.87	1.65(γ); 3.19(δ); 7.33(ε)

[a] Obtained with TSP (δ 0.00 ppm) as reference shift. Chemical shifts are accurate to ±0.02 ppm.
[b] $^3J_{\alpha N}$ coupling constants in Hz. -Δδ/ΔT = temperature coefficients (ppb/K) calculated in the range 25-40 °C.
[c] Random coil values from: Andersen et al. FEBS Lett. 1996, 399, 47-52. Further signals: C-terminal amide group, 7.18, 7.53 ppm; palmitic acid, 2.11, 1.42, 1.22-1.14, 0.79 ppm. nd: not determined; bs: broad signal; ov: overlapped signal.

Table S4. Diagnostic Medium Range NOEs observed for [Trp[1], Leu[2]]hUT-Pep3.

1	ARG	HA	3	LEU	HN	W
1	ARG	HA	4	GLN	HN	W
1	ARG	HA	4	GLN	QB	W
3	LEU	HN	5	ARG	HN	W
3	LEU	HA	6	ALA	HN	W
4	GLN	HA	6	ALA	HN	W
8	PHE	HA	11	ALA	QB	W

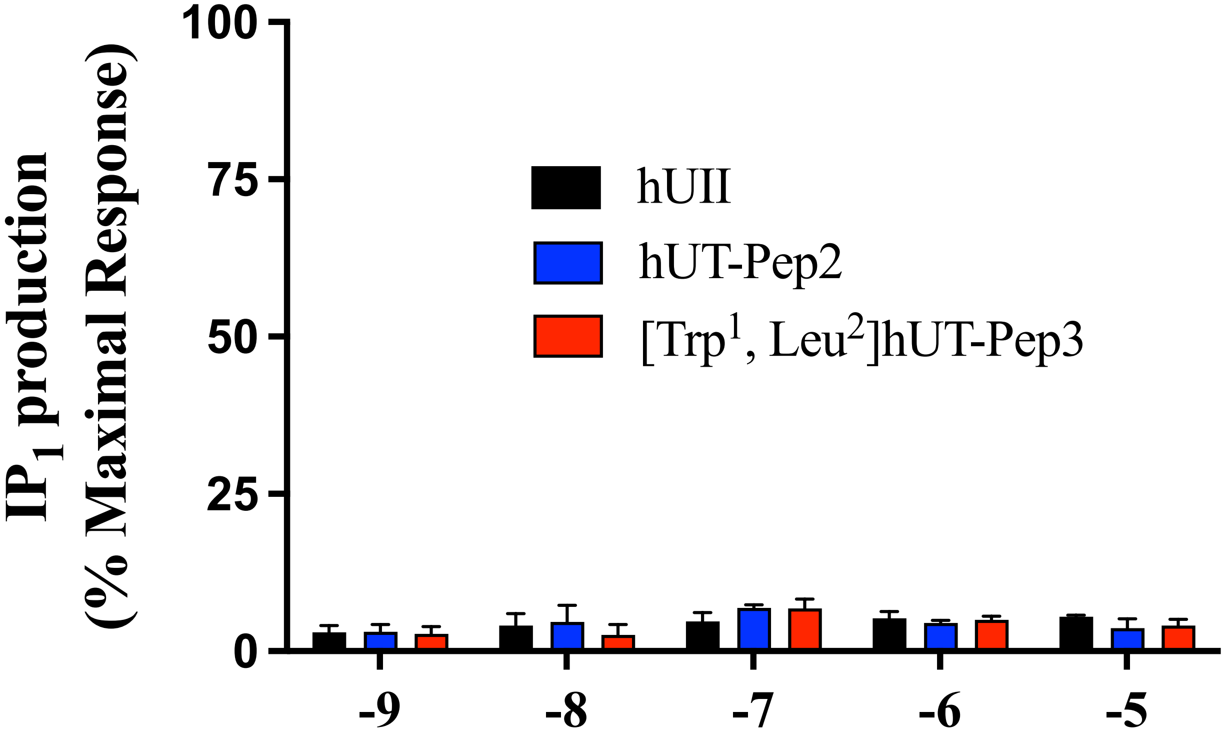

Figure S3. IP$_1$ production in CHO-K1 cells following treatment by hUII, hUT-Pep2 or [Trp^1, Leu^2]hUT-Pep3. IP$_1$ production was evaluated using the IP-One terbium immunoassay kits in CHO-K1 cells. Each histogram represents the mean ± SEM of at least three independent experiments performed in triplicate.

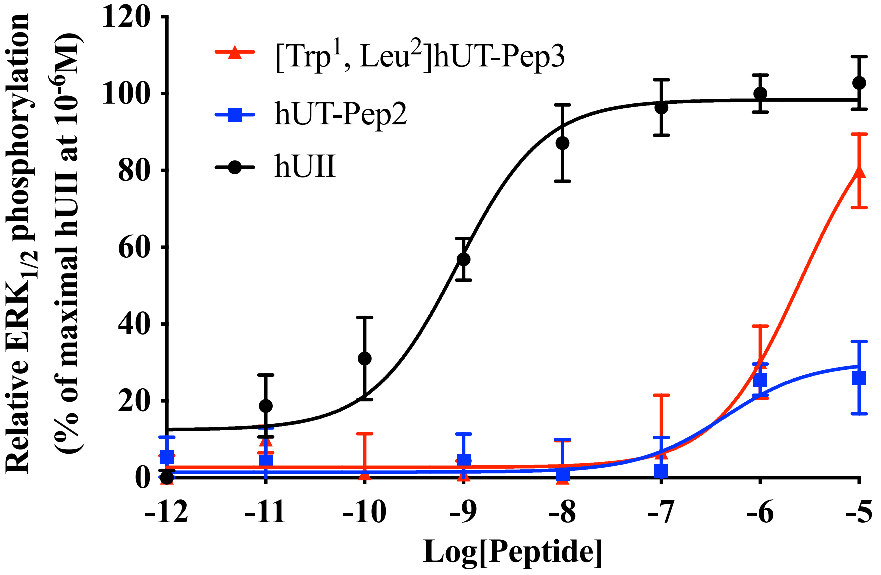

Figure S4. BRET measurements were performed following stimulation of hUT with increasing concentrations of hUII, or UT-derived pepducins in HEK 293-hUT cells. The data are normalized to maximal hUII responses (10^{-5}M). Each curve represents mean ± SEM of at least three independent concentration-response experiments performed in triplicate.

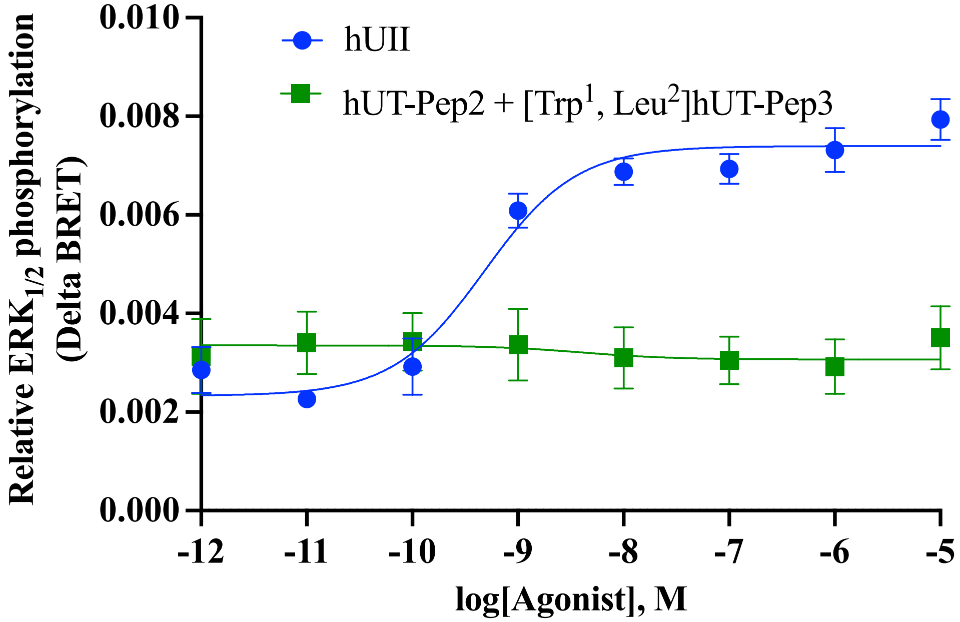

Figure S5. Effect of hUT-Pep2 and [Trp1, Leu2]hUT-Pep3 co-treatment on $ERK_{1/2}$ phosphorylation. BRET measurements were performed following hUT stimulation with increasing concentrations of hUII, or hUT-derived pepducins in HEK 293-hUT cells. Each curve represents mean ± SEM of at least three independent concentration-response experiments performed in triplicate.

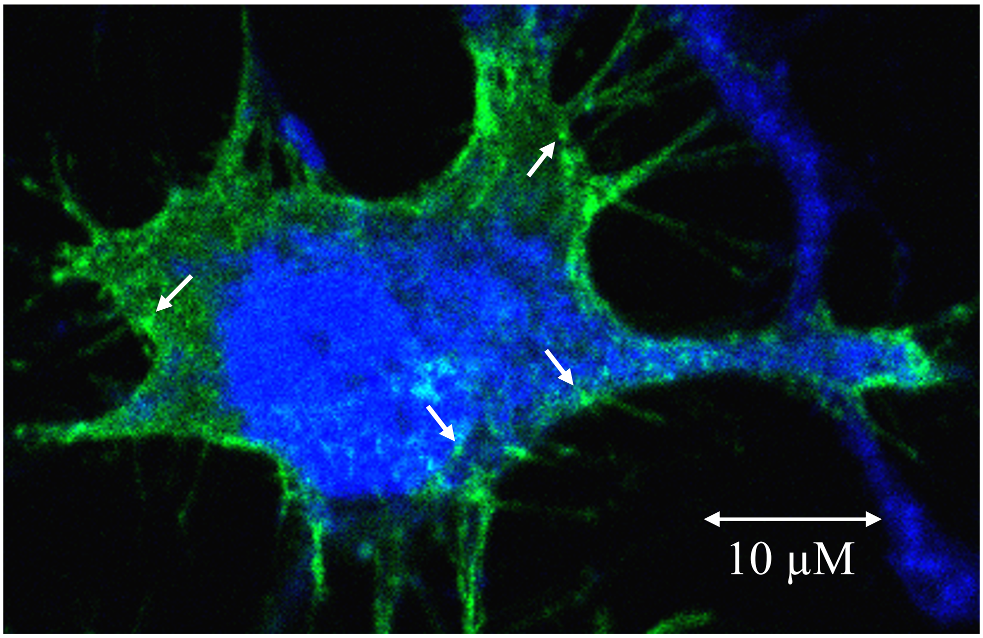

Figure S6. hUII promotes transactivation of EGFR following endogenously expressed hUT activation in untransfected HEK 293 cells. HEK 293 expressing low level of wild type hUT and transfected with GFP-EGFR were treated with hUII for 30 min prior visualization using confocal microscopy. The image shown are from representative experiments repeated at least three times. Arrows indicate cellular aggregates representing EGFR internalization.
